# PCSK9 Inhibitor Use and the Risk of Age-Related Macular Degeneration in Patients with Atherosclerotic Cardiovascular Disease

**DOI:** 10.3390/ph19050750

**Published:** 2026-05-11

**Authors:** Hou-Ren Tsai, Ji-Ze Hsu, Ching-Hui Loh, Huei-Kai Huang

**Affiliations:** 1 Department of Ophthalmology, Hualien Tzu Chi Hospital, Buddhist Tzu Chi Medical Foundation, Hualien 970, Taiwan; dr.hourentsai@gmail.com; 2 School of Medicine, Tzu Chi University, Hualien 970, Taiwan; twdoc1960@gmail.com; 3 Center for Clinical Epidemiology and Biostatistics, Hualien Tzu Chi Hospital, Buddhist Tzu Chi Medical Foundation, Hualien 970, Taiwan; 110324104@gms.tcu.edu.tw; 4 Center for Aging and Health, Hualien Tzu Chi Hospital, Buddhist Tzu Chi Medical Foundation, Hualien 970, Taiwan; 5 Department of Family Medicine, Hualien Tzu Chi Hospital, Buddhist Tzu Chi Medical Foundation, Hualien 970, Taiwan

**Keywords:** age-related macular degeneration, PCSK9 inhibitor, atherosclerotic cardiovascular disease

## Abstract

**Background/Objectives**: Emerging evidence suggests that alterations in lipid metabolism may play a contributing role in the pathogenesis of age-related macular degeneration (AMD). Proprotein convertase subtilisin/kexin type 9 (PCSK9) inhibitors, a novel class of lipid-lowering agents, offer anti-inflammatory and antioxidant benefits, which may provide protective effects against AMD. We aimed to evaluate the risk of developing AMD among patients with atherosclerotic cardiovascular disease (ASCVD) who were newly treated with PCSK9 inhibitors compared with those receiving statins. **Methods**: This retrospective cohort study utilized data from the Global Collaborative Network within the TriNetX Research Network. Patients with ASCVD who were newly initiated on PCSK9 inhibitors or statins were identified and matched for age, sex, race, laboratory data, comorbidities, and concomitant medications. The primary outcomes were the hazard ratios (HRs) for developing AMD, dry AMD, and wet AMD. Propensity score matching (PSM) was used to adjust for baseline demographics and comorbidities. **Results**: After PSM, 50,102 patients were included in each group (PCSK9 inhibitor users vs. statin users). Compared to statin users, PCSK9 inhibitor users had significantly lower risks of AMD (HR, 0.81; 95% confidence interval [CI], 0.72–0.92) and dry AMD (HR, 0.78; 95% CI, 0.65–0.94), but not wet AMD (HR, 0.90; 95% CI, 0.70–1.16). Stratified and subgroup analyses showed reduced AMD risk among patients aged ≥65 years, White patients, female patients, and evolocumab users. **Conclusions**: In patients with ASCVD, compared with use of statins, use of PCSK9 inhibitors is associated with reduced risks of AMD and dry AMD, suggesting a potential novel strategy for managing a condition with limited therapeutic options.

## 1. Introduction

Despite advances in evidence-based therapies, atherosclerotic cardiovascular disease (ASCVD) remains the leading cause of global morbidity and mortality [1,2]. A causal link exists between low-density lipoprotein cholesterol (LDL-C) and ASCVD. Interventions targeting LDL-C, particularly statins, have demonstrated safety and efficacy in reducing major cardiovascular events [3]. Monoclonal antibodies targeting proprotein convertase subtilisin/kexin type 9 (PCSK9) represent a newer class of lipid-lowering agents that significantly lower LDL-C levels [4]. Clinical studies have demonstrated that PCSK9 inhibitors reduce ischemic cardiovascular events both as monotherapy and in combination with statins [5,6].

Age-related macular degeneration (AMD) is the primary cause of vision impairment among adults aged ≥50 years [7,8], with a global prevalence of 196 million in 2020, which is projected to rise to 288 million by 2040. Increasing evidence suggests that AMD poses a significant public health challenge in aging populations [7]. A growing body of evidence suggests an association between AMD and various cardiovascular diseases (CVDs) such as acute myocardial infarction, stroke, and heart failure [9,10,11]. Atherosclerosis has long been hypothesized as a shared pathophysiological mechanism, potentially involving oxidation-specific epitopes, consequent innate immune responses, pathological neovascularization, and lipid accumulation in Bruch’s membrane [12,13,14,15]. Dysregulated lipid metabolism plays a central role in AMD pathogenesis by promoting the accumulation of lipid-rich deposits beneath the retinal pigment epithelium (RPE) and contributing to visual decline [16]. PCSK9 inhibitors exhibit pleiotropic effects, such as reducing inflammatory cytokines, oxidative stress, and apoptosis, while improving endothelial function, which may offer therapeutic benefits for AMD [17,18,19]. However, few studies have directly investigated the role of PCSK9 inhibitors in preventing AMD.

Given the increasing prevalence of ASCVD within the aging demographic, a population also susceptible to AMD development, investigating the potential pleiotropic effects of PCSK9 inhibitors on AMD risk is a pertinent clinical concern. Therefore, in this study, we aimed to evaluate the risk of developing AMD among patients with ASCVD undergoing treatment with PCSK9 inhibitors.

## 2. Results

**Figure 1:**
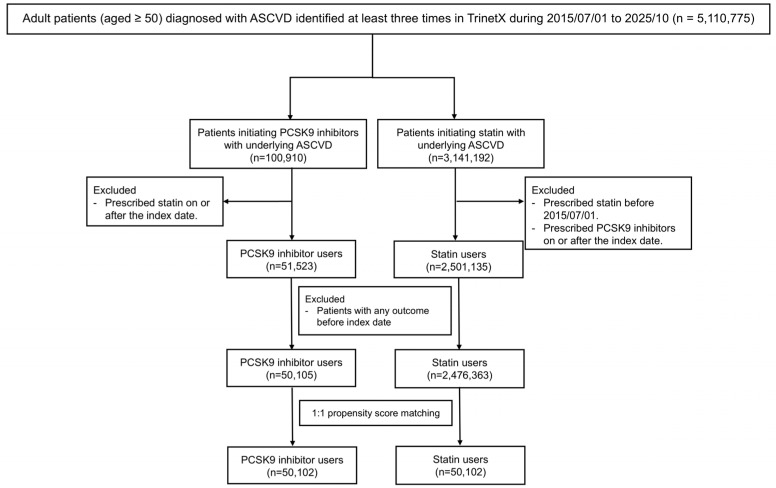
Flow diagram of the patient enrollment process. Abbreviations: ASCVD, atherosclerotic cardiovascular disease; PCSK9, proprotein convertase subtilisin/kexin type 9.

We initially identified 5,110,775 patients with ASCVD from the TriNetX dataset. After applying the inclusion and exclusion criteria, 50,102 patients were finally included in the PCSK9 inhibitor user and statin user groups (Figure 1). The mean age was 68.1 years in the PCSK9 inhibitor user group and 67.9 years in the statin user group, with female patients comprising 47.0% and 47.4% of each group, respectively. After propensity score matching (PSM), all baseline characteristics were well-balanced between the study groups (Table 1). Baseline characteristics of the study groups before PSM are shown in Table S1.

### 2.1. Risk of AMD

**Figure 2:**
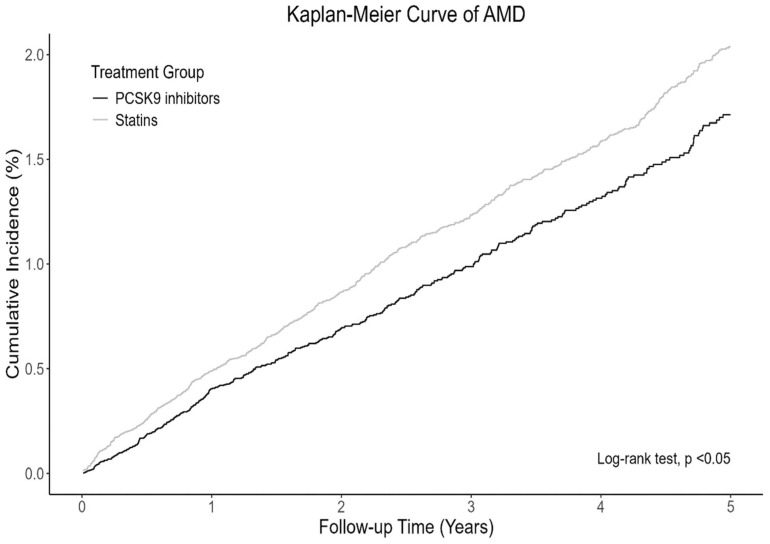
Cumulative incidence curve of AMD based on Cox proportional hazards models in patients receiving PCSK9 inhibitors or statins after PSM. Abbreviations: AMD, age-related macular degeneration; PCSK9, proprotein convertase subtilisin/kexin type 9; PSM, propensity score matching.

Overall, 397 PCSK9 inhibitor users and 691 statin users developed AMD (Table 2). The mean follow-up durations for the PCSK9 inhibitor and statin user groups were 2.36 (standard deviation [SD]: 1.67) and 3.34 (SD: 1.72) years, respectively. PCSK9 inhibitor users had a significantly lower risk of developing AMD (hazard ratio [HR]: 0.81; 95% confidence interval [CI]: 0.72–0.92) than statin users did. Figure 2 illustrates the cumulative incidence curve of the PCSK9 inhibitor user and statin user groups. Regarding individual outcome analyses, PCSK9 inhibitor use was associated with a lower risk of dry AMD (HR: 0.78; 95% CI: 0.65–0.94) but not wet AMD (HR: 0.90; 95% CI: 0.70–1.16) (Table 2).

### 2.2. Stratified and Subgroup Analyses

**Figure 3:**
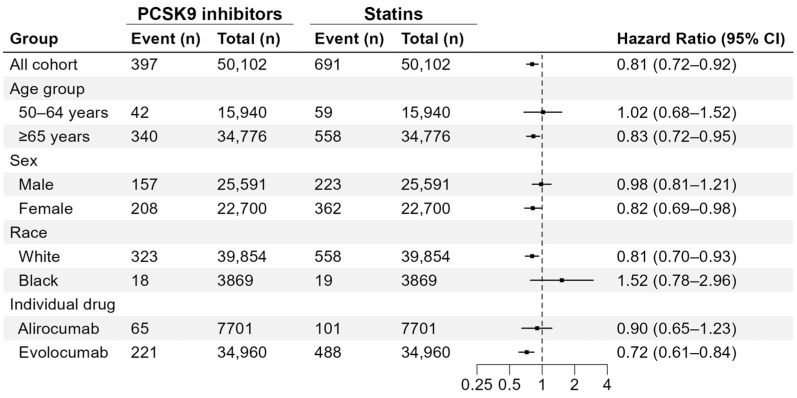
Results from stratified and subgroup analyses. Abbreviations: CI, confidence interval; PCSK9, proprotein convertase subtilisin/kexin type 9.

The results of the stratified and subgroup analysis are shown in Figure 3. By age group, PCSK9 inhibitor use was significantly associated with reduced AMD risk among patients aged ≥65 years (HR: 0.83; 95% CI: 0.72–0.95) but not among those aged 50–64 years (HR: 1.02; 95% CI: 0.68–1.52). By sex, PCSK9 inhibitor use was significantly associated with a reduced risk of AMD among female patients (HR: 0.82; 95% CI: 0.69–0.98) but not among male patients (HR: 0.98; 95% CI: 0.81–1.21). When stratified by race, the association of PCSK9 inhibitor use with a reduced risk of AMD was significant among White patients (HR: 0.81; 95% CI: 0.70–0.93) but not among African American patients (HR: 1.52; 95% CI: 0.78–2.96). Among individual drugs, evolocumab use was associated with lower AMD risk (HR: 0.72; 95% CI: 0.61–0.84), whereas alirocumab was not (HR: 0.90; 95% CI: 0.65–1.23).

### 2.3. Sensitivity Analyses

In the sensitivity analyses restricted to patients with ASCVD aged ≥60 years, PCSK9 inhibitor users exhibited a reduced risk of AMD (HR: 0.85; 95% CI: 0.74–0.97), compared with statin users. A similar trend of a protective effect was found for dry AMD (HR: 0.87; 95% CI: 0.71–1.06); however, no significant association was observed for wet AMD (HR: 1.15; 95% CI: 0.88–1.50). Among patients aged ≥70 years, similar results were observed (AMD HR: 0.81; 95% CI: 0.70–0.93; dry AMD HR: 0.77; 95% CI: 0.62–0.95; wet AMD HR: 1.06; 95% CI: 0.80–1.41; Table S2).

## 3. Discussion

In this large cohort study, patients initiated on PCSK9 inhibitors demonstrated a lower risk of developing AMD, compared with those newly treated with statins. Our sensitivity analyses results remained consistent when restricted to patients with ASCVD aged ≥60 years (or ≥70 years).

Although the precise mechanisms remain unclear, previous studies provide potential explanations. First, pathological studies have shown lipid accumulation in Bruch’s membrane in AMD, resembling atherosclerotic changes in CVD [20,21,22,23]. More than 40% of drusen—the hallmark histopathologic feature of AMD—consists of lipids, including esterified cholesterol, unesterified cholesterol, and phosphatidylcholine [24]. PCSK9, a key regulator of LDL-C metabolism, may play a role in mitigating AMD. One case report [25] documented improvement in pachydrusen during PCSK9 inhibitor therapy, with subsequent worsening after discontinuation. Considering that PCSK9 proteins circulate systemically, including in the vitreous fluid of eyes with retinal detachment, they likely reach areas of high blood flow, such as the choroid [26]. Second, although AMD ultimately causes neurodegeneration, it likely originates in the choroidal vasculature. The vascular model of macular degeneration suggests that impaired choroidal perfusion of the RPE leads to lipoprotein accumulation in drusen and Bruch’s membrane [27]. Additionally, retinal microcirculation in AMD is influenced by genetic factors, choroidal characteristics, and clinical determinants [28]. Disruptions in choroidal and retinal blood flow are critical to AMD progression. PCSK9 inhibitors have been shown to improve retinal microvascular function and reduce small artery stiffness, which may benefit diseases such as AMD that are influenced by microvascular health [29]. Lastly, protective effects of PCSK9 inhibitors against inflammation have been demonstrated in rat retinal Müller cells [30]. This anti-inflammatory effect is partly mediated through the negative regulation of the Toll-like receptor 4 and nuclear factor-kappa B signaling pathway, a mechanism that may be relevant to AMD [31,32]. By improving lipid profiles and mitigating retinal vascular dysfunction, oxidative stress, and inflammation, PCSK9 inhibitors may reduce the risk of AMD.

Our findings indicate that PCSK9 inhibitors are associated with a reduced risk of dry AMD but not wet AMD, and this pattern was consistent in sensitivity analyses among individuals aged ≥60 and ≥70 years. However, the direction of the association was similar across AMD subtypes. The non-significant finding for wet AMD is likely due to the smaller number of events, resulting in limited statistical power and wider confidence intervals. Therefore, these findings should not be interpreted as conclusive evidence of a differential effect between AMD subtypes. Further studies with larger sample sizes are warranted to confirm this association. Our findings align with a recent study demonstrating the protective effect of PCSK9 inhibitors against AMD risk, particularly the dry subtype. Of note, our current study differs from this prior work in three main aspects [33]. First, we evaluated patients with established ASCVD instead of general dyslipidemia, a population chosen due to the potential shared mechanisms between cardiovascular conditions and AMD [34]. Second, our analysis utilized a different set of covariates to account for potential confounding factors. Third, we included stratified analyses to further explore these associations within specific clinical subgroups. These methodological differences may provide supplementary insights that add to the existing literature.

Age- and sex-stratified analyses revealed numerically lower AMD risks across all comparisons, with a significant association observed in individuals aged ≥65 years and female patients. Given the higher risks of AMD in older and female patients [35], the effect of PCSK9 inhibitors may be more pronounced in groups with a higher disease burden. Also, previous studies indicate that PCSK9 concentrations fluctuate with the menstrual cycle and menopausal status, suggesting a potential regulatory role for estrogen [36,37,38]. This hormonal influence is hypothesized to be mediated by the activation of G-protein-coupled estrogen receptors on hepatocytes [36,37]. Given that our female cohort consists entirely of individuals over 50 years of age, they are presumed to largely represent a postmenopausal demographic. Consequently, we speculate that the physiological decline in estrogen levels following menopause might contribute to elevated PCSK9 expression, which could potentially explain the pronounced effects observed in these women [38]. Further research exploring potential sex-specific differences is needed to evaluate the generalizability of these initial observations.

The racial distribution suggests that AMD is most common in White populations, with studies reporting prevalences of 5.4, 4.6, and 2.4% in Whites, Asians, and African Americans, respectively [39]. The higher prevalence of AMD in White populations may lead to a more pronounced reduction in AMD risk among PCSK9 inhibitor users of these races. On the other hand, racial differences in the use of lipid-lowering treatments have been documented, and prior research [40] indicates that African American patients may be less likely to trust the effectiveness of these medications, which might partly explain the opposite, although not statistically significant, trend observed in this group. However, because PCSK9 inhibitors are relatively new, there is still limited evidence on patient adherence to and access to these therapies. Genetic differences may also play a role in the observed outcome. As indicated by previous research [41], there are differences in PCSK9 levels and activity among different ethnicities. However, further multi-ethnic studies are still needed to confirm the findings. Notably, although we utilized the Global Collaborative Network for our analysis, patients of Asian descent comprised less than 5% of the population within the TriNetX Research Network. This limited sample size precluded meaningful stratified analyses for this specific subgroup.

To date, few studies have examined the association between PCSK9 inhibitors and AMD risk; our study addresses this gap. The main strengths of this study include the use of a large-scale cohort, as well as novel findings linking PCSK9 inhibitors to reduced AMD risk. Sensitivity analyses reinforce the robustness of our results. However, some limitations of this study must be acknowledged. First, despite the vast scale of the TriNetX database, it inherently lacks granular data on critical lifestyle and clinical variables, including dietary habits, tobacco use, and physical activity. Because these are established risk factors for AMD, their absence leaves the analysis susceptible to residual confounding. Second, the database does not provide details on medication dosage, duration, or frequency, limiting assessment of treatment intensity. Thirdly, reliance on diagnostic codes may lead to misclassification of exposures and outcomes. However, this misclassification is likely to be non-differential across treatment groups, which would tend to bias effect estimates toward the null [42]. Lastly, a notable limitation of this study is the shorter follow-up duration in the PCSK9 inhibitor group compared with the statin group, which may reflect the more recent clinical adoption of these agents. Given the slow progression of AMD, shorter follow-up may lead to under-ascertainment of events. However, time-to-event analyses utilized in this study account for varying follow-up durations and right-censoring, and thus this difference is unlikely to have substantially biased the estimated associations. Further studies with longer follow-up durations are warranted to better capture the long-term risk of AMD.

## 4. Materials and Methods

### 4.1. Study Design and Data Sources

This retrospective cohort study was conducted using data from the Global Collaborative Network within the TriNetX Research Network (Cambridge, MA, USA) [43]. TriNetX collects deidentified electronic health record data across healthcare organizations, primarily those based in the United States, as well as locations in Australia, the United Kingdom, Spain, Bulgaria, India, Malaysia, and Taiwan. The network includes hospitals, primary care providers, and specialists who contribute data from both insured and uninsured patients. TriNetX offers access to deidentified electronic health records from more than 250 million individuals across over 120 healthcare organizations worldwide. Detailed information about TriNetX is available on their website: https://trinetx.com/ (accessed on 4 November 2025). Data quality within TriNetX is assessed using standardized criteria that evaluate metrics such as conformance, completeness, and plausibility [44]. The study adhered to the Strengthening the Reporting of Observational Studies in Epidemiology guidelines for cohort studies. The research ethics committee at Hualien Tzu Chi Hospital gave its approval for the study protocol (REC number: IRB114-194-C).

### 4.2. Study Population and Exposure

Adults aged ≥50 years with ASCVD between July 2015 and October 2025 were included. As the FDA first approved the PCSK9 inhibitor evolocumab in 2015, the enrollment period for participants with ASCVD began in July 2015 [45]. ASCVD was defined as at least three separate diagnoses of ischemic heart disease (International Classification of Diseases, 10th Revision [ICD-10] code: I20–I25), cerebrovascular disease (ICD-10 code: I60–I69), or peripheral artery disease (ICD-10 code: I73) during the study period. The first cohort, the “PCSK9 inhibitor users,” comprised individuals prescribed PCSK9 inhibitors alirocumab (RxNorm code: 1659152), evolocumab (RxNorm code: 1665684), and inclisiran (RxNorm code: 2588243), as classified by the National Library of Medicine’s normalized naming system for generic and branded drugs. For this group, the initiation of PCSK9 inhibitor therapy marked the index date. Patients who used statins after the index date (switchers) were excluded. In contrast, the cohort labelled “statin users” included individuals who were newly prescribed statins (Anatomical Therapeutic Chemical code C10AA). This cohort included ASCVD patients initiating statin therapy after July 2015, with no prior statin prescriptions, and the date of this initial prescription served as their index date. To prevent confounding, we excluded patients who subsequently used PCSK9 inhibitors (switchers). Furthermore, to ensure the evaluation of only incident cases, individuals diagnosed with AMD prior to their respective index dates were excluded from both the exposed and nonexposed cohorts.

### 4.3. Outcome Measures

The primary outcome was incident AMD, identified using the ICD-10 codes H35.30, H35.31, and H35.32. Tracking of all study participants began at their respective index dates and continued until the occurrence of AMD, death, or the end of available date in the databases, whichever occurred first. Additionally, individual outcome analyses were conducted for dry AMD (ICD-10 code: H35.31) and wet AMD (ICD-10 code: H35.32). In these analyses, patients were followed up until the specific outcome occurred; for example, a patient diagnosed with dry AMD who later developed wet AMD was also included in the wet AMD analysis.

### 4.4. Covariates and Confounders

The baseline characteristics of eligible patients were extracted from the TriNetX dataset and included age, sex, race, ethnicity, healthcare utilization, lifestyle factors, comorbidities, and baseline medication use. Pre-existing comorbidities and medication use at baseline (Table S3), identified as potential confounders, were selected based on prior studies [46,47]. A comorbidity was considered present if a diagnosis had been recorded within 1 year before the index date. Baseline medication use was defined as any recorded prescription within 1 year before the index date.

### 4.5. Statistical Analyses

All statistical analyses were conducted using the TriNetX Analytics network platform and R software version 4.4.1 (R Foundation for Statistical Computing, Vienna, Austria). Systematic differences in the baseline characteristics and potential confounders between the exposed and unexposed cohorts were addressed through PSM following cohort definition. Multivariable logistic regression models incorporating covariates listed in Table 1 were used to calculate the propensity scores, estimating each patient’s likelihood of receiving PCSK9 inhibitors or statins. Nearest-neighbor matching without replacement was applied using a caliper width of 0.2 standard deviations of the logit of the propensity score [48,49]. Before any data analysis, including overall, subgroup, and stratified analyses, PSM was performed separately for each comparison set. Standardized mean differences were used to assess differences in baseline characteristics between study cohorts, with values <0.1 indicating negligible differences [50]. The Kaplan–Meier method was used to plot cumulative incidence curves, and the log-rank test was employed to compare time-to-event outcomes. The HRs for each outcome were estimated using Cox proportional hazards models. The proportional hazards assumption was tested using the generalized Schoenfeld method integrated into the TriNetX platform, with no violations detected [51].

Stratified analyses were performed by age group (50–64 and ≥65 years), sex (male and female), and race (White, African American, and Asian) to explore potential effect modification. Based on the first prescribed type of PCSK9 inhibitors, the exposed cohort was further classified into two subgroups: evolocumab and alirocumab users. These subgroups were compared with the statin cohort to estimate the relative effect of each PCSK9 inhibitors on AMD risk. A two-tailed *p*-value < 0.05 was considered statistically significant.

Sensitivity analyses were conducted to assess the robustness of the findings. Given the age-related increase in AMD risk, we limited the cohort to older patients (≥60 and ≥70 years) to examine the effects in the most relevant demographic [7].

## 5. Conclusions

Our analysis reveals a protective association between PCSK9 inhibitor use and the incidence of AMD–particularly dry AMD–among patients with ASCVD compared to statin users. Considering the limited therapeutic options for dry AMD, this potential association highlights an interesting avenue for future research into the secondary effects of lipid-lowering therapies.

## Figures and Tables

**Table 1:** Demographic data of the study population after propensity score matching.

Characteristics *			
	PCSK9 Inhibitor Users (*n* = 50,102)	Statin Users (*n* = 50,102)	SMD ^†^
Age at the index date	68.1 ± 8.4	67.9 ± 8.9	0.027
Sex, n (%)			
Male	26,489 (52.9)	26,311 (52.5)	0.007
Female	23,563 (47.0)	23,748 (47.4)	0.007
Unknown sex	50 (0.1)	43 (0.1)	0.005
Ethnicity, n (%)			
Not Hispanic or Latino	40,538 (80.9)	40,943 (81.7)	0.021
Hispanic or Latino	1408 (2.8)	1251 (2.5)	0.019
Unknown ethnicity	8156 (16.3)	7908 (15.8)	0.013
Race, n (%)			
White	41,340 (82.5)	42,327 (84.5)	0.053
African American	3874 (7.7)	3514 (7.0)	0.027
Asian	1209 (2.4)	1105 (2.2)	0.014
American Indian or Alaska Native	173 (0.3)	170 (0.3)	0.001
Native Hawaiian or Other Pacific Islander	121 (0.2)	131 (0.3)	0.004
Other race	885 (1.8)	818 (1.6)	0.010
Unknown race	2500 (5.0)	2037 (4.1)	0.044
Comorbidities, n (%)			
Hypertension	32,322 (64.5)	31,934 (63.7)	0.016
Diabetes mellitus	14,333 (28.6)	13,454 (26.9)	0.039
Heart failure	7300 (14.6)	6591 (13.2)	0.041
Atrial fibrillation and flutter	6797 (13.6)	6387 (12.7)	0.024
Hypermetropia	365 (0.7)	332 (0.7)	0.008
Chronic kidney disease	6563 (13.1)	6032 (12.0)	0.032
Lifestyle			
Tobacco use, n (%)	1551 (3.1)	1388 (2.8)	0.019
Laboratory data			
BMI	30.3 ± 6.1	30.6 ± 6.8	0.041
BMI ≥ 30 kg/m^2^, n (%)	18,261 (36.4)	18,238 (36.4)	0.001
Triglyceride	169.2 ± 149.5	173.0 ± 140.2	0.026
HDL	48.0 ± 17.4	47.1 ± 19.1	0.051
LDL	123.5 ± 50.4	122.8 ± 46.0	0.016
LDL ≥ 130 mg/dL, n (%)	15,509 (31.0)	15,269 (30.5)	0.010
Total cholesterol	204.5 ± 58.9	203.6 ± 56.8	0.016
Medical service utilization, n (%)			
Office or other outpatient services	27,270 (54.4)	27,345 (54.6)	0.003
Emergency department services	9413 (18.8)	8759 (17.5)	0.034
Hospital inpatient and observation care services	7643 (15.3)	6859 (13.7)	0.044
Medication, n (%)			
Corticosteroid	17,831 (35.6)	17,800 (35.5)	0.001
Non-steroidal anti-inflammatory drug	8977 (17.9)	8776 (17.5)	0.011
Metformin	4679 (9.3)	4514 (9.0)	0.011
Lutein	209 (0.4)	168 (0.3)	0.013
Zeaxanthin	25 (0.1)	30 (0.1)	0.004

* All covariates listed were used for propensity score matching. ^†^ A standardized mean difference <0.1 indicates a negligible difference. Abbreviations: BMI, body mass index; HDL, high-density lipoprotein; LDL, low-density lipoprotein; PCSK9, proprotein convertase subtilisin/kexin type 9; SMD, standardized mean difference.

**Table 2:** Risk of incident AMD among patients with ASCVD treated with PSCK9 inhibitors vs. statins.

	Patients with Outcome			
Outcomes	PCSK9 Inhibitor Users (*n* = 50,102)	Statin Users (*n* = 50,102)	HR * (95% CI)	*p*-Value for Log-Rank Test
AMD	397	691	0.81 (0.72–0.92)	0.001
Dry AMD	181	329	0.78 (0.65–0.94)	0.011
Wet AMD	98	163	0.90 (0.70–1.16)	0.444

* The HRs were calculated using a univariable Cox regression model with propensity score matching, with the corresponding statin user group as the reference. Abbreviations: AMD, age-related macular degeneration; ASCVD, atherosclerotic cardiovascular disease; CI, confidence interval; HR, hazard ratio; PCSK9, proprotein convertase subtilisin/kexin type 9.

## Data Availability

The original contributions presented in this study are included in the article and Supplementary Materials Further inquiries can be directed to the corresponding author due to TriNetX’s data protection policy.

## Supplementary Materials

The following supporting information can be downloaded at: https://www.mdpi.com/article/10.3390/ph19050750/s1, Table S1: Demographic data of the study population before propensity score matching; Table S2: Risk of incident AMD in patients aged ≥60 and ≥70 years with ASCVD treated with PCSK9 inhibitors versus statins; Table S3: Codes for covariates.

## Abbreviations

The following abbreviations are used in this manuscript:

AMD	Age-related macular degeneration
ASCVD	Atherosclerotic cardiovascular disease
CI	Confidence interval
CVD	Cardiovascular disease
HR	Hazard ratio
ICD-10	International Classification of Diseases, 10th Revision
LDL-C	Low-density lipoprotein cholesterol
PCSK9	Proprotein convertase subtilisin/kexin type 9
PSM	Propensity score matching
RPE	Retinal pigment epithelium
SD	Standard deviation
VEGF	Vascular endothelial growth factor
