# Supplementary material for: PCSK9 Inhibitor Use and the Risk of Age-Related Macular Degeneration in Patients with Atherosclerotic Cardiovascular Disease
Source: Pharmaceuticals (Basel). 2026 May 11;19(5):750. doi: 10.3390/ph19050750 (PMC13210746; doi:10.3390/ph19050750)
Supplement: Supplementary file 1 [file pharmaceuticals-19-00750-s001.zip › pharmaceuticals-4230719-supplementary.pdf]

Supplementary Materials

Table S1. Demographic data of the study population before propensity-score matching

**Characteristics**[a]	**PCSK9 inhibitor users (*n* = 50,105)**	**Statin users (*n* = 2,476,363)**	**SMD**[b]
Age at the index date	68.1±8.4	67.3±9.5	0.089
Sex, %			
Male	26,489 (52.9)	1,484,773 (60.0)	0.143
Female	23,565 (47.0)	990,791 (40.0)	0.142
Unknown Sex	51 (0.1)	799 (0.0)	0.027
Ethnicity, n (%)			
Not Hispanic or Latino	40,541 (80.9)	1,750,043 (70.7)	0.241
Hispanic or Latino	1408 (2.8)	114,967 (4.6)	0.097
Unknown ethnicity	8156 (16.3)	611,353 (24.7)	0.210
Race, n (%)			
White	41,343 (82.5)	1,696,470 (68.5)	0.330
African American	3874 (7.7)	313,717 (12.7)	0.164
Asian	1209 (2.4)	102,158 (4.1)	0.096
American Indian or Alaska Native	173 (0.3)	9941 (0.4)	0.009
Native Hawaiian or Other Pacific Islander	121 (0.2)	10,851 (0.4)	0.034
Other race	885 (1.8)	62,347 (2.5)	0.052
Unknown race	2500 (5.0)	280,879 (11.3)	0.234
Comorbidities, n (%)			
Hypertension	32,325 (64.5)	1,056,450(42.7)	0.449
Diabetes mellitus	14,336 (28.6)	550,851(22.2)	0.147
Heart failure	7303 (14.6)	271,979(11.0)	0.108
Atrial fibrillation and flutter	6799 (13.6)	254,686 (10.3)	0.101
Hypermetropia	365 (0.7)	9461 (0.4)	0.047
Chronic kidney disease	6564 (13.1)	254,496 (10.3)	0.088
Lifestyle			
Tobacco use, n (%)	1551 (3.1)	64,167 (2.6)	0.030
Laboratory data			
BMI	30.3±6.1	29.7±6.8	0.087
BMI ≥30 kg/m², n (%)	18,263 (36.4)	535,745 (21.6)	0.331
Triglyceride	169.2±149.5	144.7±118.8	0.181
HDL	48.0±17.4	47.4±18.7	0.037
LDL	123.6±50.4	103.6±42.3	0.429
LDL ≥130 mg/dL, n (%)	15,512 (31.0)	197,928 (8.0)	0.606
Total cholesterol	204.5±58.9	179.2±52.5	0.453
Medical service utilization, n (%)			
Office or other outpatient services	27,272 (54.4)	734,587 (29.7)	0.518
Emergency department services	9413 (18.8)	440,388 (17.8)	0.026
Hospital inpatient and observation care services	7644 (15.3)	285,688 (11.5)	0.109
Medication, n (%)			
Corticosteroid	17,834 (35.6)	509,082 (20.6)	0.339
Non-steroidal anti-inflammatory drug	8978 (17.9)	281,850 (11.4)	0.186
Metformin	4681 (9.3)	117,108 (4.7)	0.181
Lutein	209 (0.4)	3393 (0.1)	0.053
Zeaxanthin	25 (0.1)	575 (0.0)	0.014

[a]All covariates listed were used to calculate the propensity score for matching.
[b]A standardized mean difference <0.1 indicates a negligible difference.

Abbreviations: BMI, body mass index; HDL, high-density lipoprotein; LDL, low-density lipoprotein; PCSK9, proprotein convertase subtilisin/kexin type 9; SMD, standardized mean difference

Table S2. Risk of incident AMD among patients aged ≥60 and ≥70 years with ASCVD treated with PCSK9 inhibitors versus statins

	PCSK9 inhibitor users		Statin users			
**Outcomes**	**Events, No.**	**Total**	**Events, No.**	**Total**	**HR[a] (95% CI)**	***P*-value for log-rank test**
**Development of AMD among patients with ASCVD aged ≥60 years**						
AMD	361	41,898	598	41,898	0.85 (0.74–0.97)	0.018
Dry AMD	155	41,898	255	41,898	0.87 (0.71–1.06)	0.177
Wet AMD	96	41,898	126	41,898	1.15 (0.88–1.50)	0.299
**Development of AMD among patients with ASCVD aged ≥70 years**						
AMD	307	25,129	512	25,129	0.81 (0.70–0.93)	0.004
Dry AMD	132	25,129	232	25,129	0.77 (0.62–0.95)	0.018
Wet AMD	82	25,129	114	25,129	1.06 (0.80–1.41)	0.666

[a]The hazard ratios were calculated using a univariable Cox regression model with propensity score matching, with the corresponding statin group as the reference.

Abbreviations: AMD, age-related macular degeneration; ASCVD, atherosclerotic cardiovascular disease; CI, confidence interval; HR, hazard ratio; PCSK9, proprotein convertase subtilisin/kexin type 9.

Table S3. Codes for covariates

Baseline comorbidity	ICD-10 code
Hypertension	I10
Atrial fibrillation and flutter	I48
Diabetes mellitus	E08–E13
Chronic kidney disease	N18
Heart failure	I50
Hypermetropia	H52.0
**Baseline medication**	**Drug code**
Corticosteroid	ATC code = D07
Non-steroidal anti-inflammatory drugs	ATC code = M01A
Metformin	RxNorm 6809
Lutein	RxNorm 11359
Zeaxanthin	RxNorm 39918

Abbreviations: ATC code, Anatomical Therapeutic Chemical code; ICD-10, International Classification of Diseases, Tenth Revision
